# WELLFOCUS PPT – modified positive psychotherapy to improve well-being in psychosis: study protocol for a pilot randomised controlled trial

**DOI:** 10.1186/1745-6215-15-203

**Published:** 2014-06-03

**Authors:** Beate Schrank, Simon Riches, Tony Coggins, Tayyab Rashid, Andre Tylee, Mike Slade

**Affiliations:** 1 Health Service and Population Research Department, Institute of Psychiatry, King’s College, London, United Kingdom; 2 South London and Maudsley NHS Foundation Trust, Mental Health Promotion, London, United Kingdom; 3 Health & Wellness Centre, University of Toronto, Toronto, Canada; 4 Department of Psychiatry and Psychotherapy, Medical University of Vienna, Vienna, Austria

**Keywords:** Intervention, Mental illness, Positive psychology, Positive psychotherapy, Psychosis, Randomised controlled trial, Recovery, Well-being

## Abstract

**Background:**

The promotion of well-being is an important goal of recovery oriented mental health services. No structured, evidence-based intervention exists that aims to increase the well-being in people with severe mental illness such as psychosis. Positive psychotherapy (PPT) is a promising intervention for this goal. Standard PPT was adapted for use with people with psychosis in the UK following the Medical Research Council framework for developing and testing complex interventions, resulting in the WELLFOCUS Model describing the intended impact of WELLFOCUS PPT. This study aims to test the WELLFOCUS Model, by piloting the intervention, trial processes, and evaluation strategy.

**Methods/Design:**

This study is a non-blinded pragmatic pilot RCT comparing WELLFOCUS PPT provided as an 11-session group therapy in addition to treatment as usual to treatment as usual alone. Inclusion criteria are adults (aged 18–65 years) with a main diagnosis of psychosis who use mental health services. A target sample of 80 service users with psychosis are recruited from mental health services across the South London and Maudsley NHS Foundation Trust. Participants are randomised in blocks to the intervention and control group. WELLFOCUS PPT is provided to groups by specifically trained and supervised local therapists and members of the research team. Assessments are conducted before randomisation and after the group intervention. The primary outcome measure is well-being assessed by the Warwick-Edinburgh Mental Well-being Scale. Secondary outcomes include good feelings, symptom relief, connectedness, hope, self-worth, empowerment, and meaning. Process evaluation using data collected during the group intervention, post-intervention individual interviews and focus groups with participants, and interviews with trial therapists will complement quantitative outcome data.

**Discussion:**

This study will provide data on the feasibility of the intervention and identify necessary adaptations. It will allow optimisation of trial processes and inform the evaluation strategy, including sample size calculation, for a future definitive RCT.

**Trial registration:**

Current Controlled Trials ISRCTN04199273 – WELLFOCUS study: an intervention to improve well-being in people with psychosis, Date registered: 27 March 2013, first participant randomised on 26 April 2013.

## Background

Developing a recovery orientation in mental health services is a policy goal in the UK and internationally [1]. Recovery is an individual process of gradual restoration from the illness, focusing on the resources and abilities of the individual instead of exclusively treating symptoms. Hence, supporting recovery from severe mental illness involves, in addition to treating symptoms, an increased emphasis on personal strengths [2], positive identity development [3], and the promotion of well-being [4].

Well-being is not only a central component of recovery from mental illness [5], its importance is further supported by research showing an association between well-being and improved functioning [6,7], increased resilience and life satisfaction [6], and suggesting its protective value against the onset or re-occurrence of mental illness [8,9]. There is strong evidence that well-being is not only a desirable outcome in its own right, but also a statistically significant predictor of symptomatic response in the treatment of people with psychosis [10,11] and strongly associated with medication compliance in this group [12]. Despite the relevance of well-being research to recovery [13], no structured intervention based on an empirically-defensible theory exists which targets well-being in people with severe mental illnesses such as psychosis [14].

The promotion of well-being is a focus of the academic discipline of positive psychology; one intervention based on this body of knowledge is positive psychotherapy (PPT). PPT was originally developed for depression as the target condition, following the hypothesis that depression can not only be treated effectively by reducing its negative symptoms but also by directly and primarily building positive emotions, character strengths, engagement, and meaning [15].

Evidence from intervention studies suggests that standard PPT is beneficial for people suffering distress or common mental disorders. Randomised controlled trials (RCTs) comparing PPT with no treatment show increased positive affect and life satisfaction and decreased depressive symptoms in undiagnosed students [16], in a self-referred online sample [8], in undiagnosed middle school students [17], and in students with a diagnosis of mild to moderate or severe depression [15,18]. PPT also showed statistically significant well-being gains and depressive symptom relief as compared to placebo therapy (e.g., recording early memories) in RCTs involving students with mild to moderate depression and in an undiagnosed community sample [15,19]. Further small studies from Iran (RCT) and Chile (quasi-experimental control group design) comparing PPT to behavioural therapy in patients with diagnosed depression showed PPT to be more effective in increasing happiness and decreasing depression [20,21]. Moreover, a meta-analysis of RCT evidence demonstrated that positive psychology interventions in general, including mindfulness, positive writing, hope, gratitude, forgiveness, kindness therapies, Fordyce’s Happiness Program, and PPT, statistically significantly improve well-being and decrease depressive symptoms for people with depression [22]. A dose–response effect was also present, with positive effect increasing with the number of different positive exercises in a therapy programme. Recently, a small uncontrolled feasibility study of PPT yielded promising results for people with psychosis in the US, statistically significantly increasing participants well-being, savouring beliefs, hope, self-esteem, and personal recovery scores [9].

**Figure 1:**
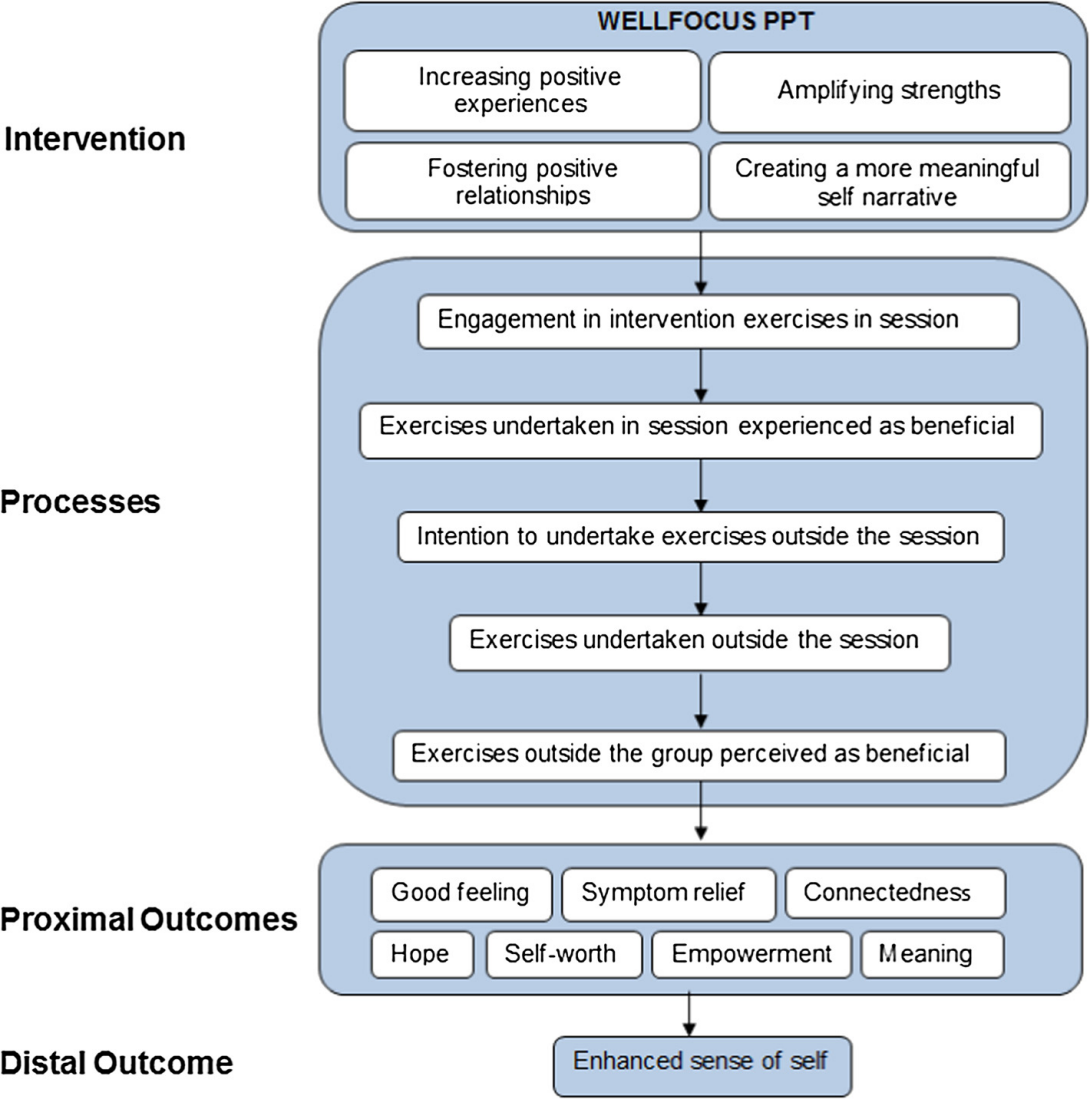
The WELLFOCUS model.

Based on the above preliminary evidence, we adapted standard PPT [23] for use with people with psychosis in a UK context. The scientific framework for the study is the Medical Research Council (MRC) Framework for Evaluating Complex Health Interventions [24]. This involved a systematic review [14], a qualitative study [25], and an expert consultation to adapt the intervention and develop an intervention model to be tested in the present pilot RCT.The new intervention, i.e., WELLFOCUS PPT, has four target areas: increasing positive experiences, amplifying strengths, fostering positive relationships, and creating a more meaningful self-narrative. The WELLFOCUS model describes the intended impact of WELLFOCUS PPT, and is shown in Figure 1.

### Aims

The aim of this pilot RCT is to test the WELLFOCUS model. The three objectives are:

#### Objective 1: Piloting the intervention

To identify whether WELLFOCUS PPT is feasible and acceptable and determine any necessary modifications.

#### Objective 2: Piloting the trial processes

To test procedures for a future definitive RCT, especially in relation to eligibility criteria, randomisation procedures, allocation processes, and recruitment and retention rates.

#### Objective 3: Piloting the evaluation strategy

To test approaches to assessing fidelity, process evaluation, and outcome evaluation, to inform the design of a future definitive RCT including choice of primary and secondary outcome measures, and sample size calculation.

## Methods

### Hypotheses

While we shall conduct hypothesis testing, this is not the main objective of the study, as this is the purpose of the future definitive RCT. The reporting of the study will not therefore emphasise the results of hypothesis testing over the reporting of whether the objectives were met [26].Our hypotheses are derived from the WELLFOCUS Model shown in Figure 1. Regarding the effect of the intervention, we hypothesise that participants receiving the intervention will experience, compared to the control group, an improvement in well-being (primary outcome) and good feelings, symptoms, connectedness, hope, self-worth, empowerment, and meaning in life (secondary outcomes).

### Design

**Figure 2:**
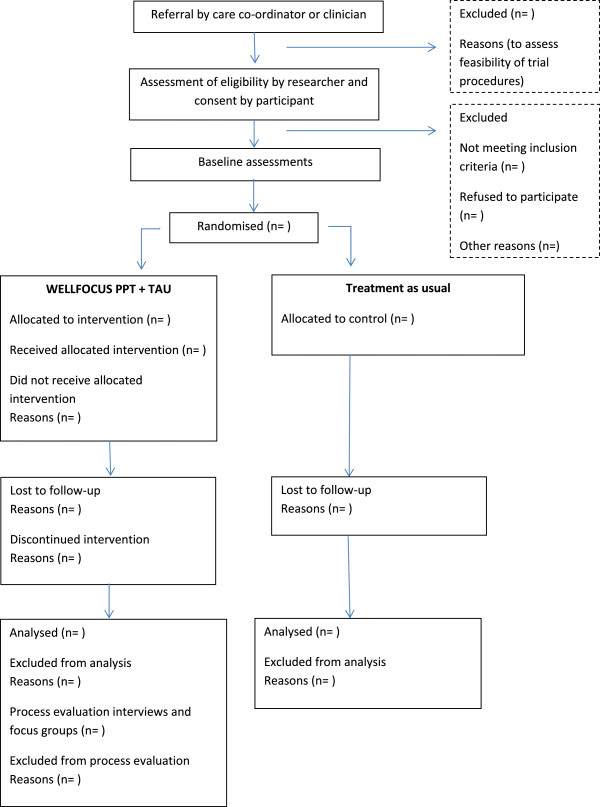
Consort flow chart of WELLFOCUS RCT.

This is a single centre pilot RCT to test WELLFOCUS PPT in a group format in a convenience sample of people with psychosis. Patients are block-randomised to receive either WELLFOCUS PPT in addition to treatment as usual, or to continue to receive treatment as usual only. The design of this pilot RCT has been informed by recommendations for the conduct of pilot trials by Tabane et al. [27] and Lancaster et al. [28]. The Consort flow diagram [29] is shown in Figure 2.

### Ethical approval

Ethical approval for the WELLFOCUS trial was obtained from the Camberwell St Giles Research Ethics Committee (reference 12/LO/1960). R&D approval was obtained from South London and Maudsley NHS Foundation Trust. Participants receive verbal and written information, and written informed consent is obtained from participants before they enter the study. The research is conducted in compliance with the Declaration of Helsinki [30].

### Study setting

Patients are recruited from seven teams and two research registers across the South London and Maudsley NHS Foundation Trust (SLaM). SLaM employs 4,500 staff in 296 teams, works with 34,128 service users, and provides adult mental health services across four Boroughs (Croydon, Lambeth, Lewisham, and Southwark). These services are provided through Clinical Academic Groups organised according to diagnoses; these groups bring together clinical services, research, education, and training for the benefit of patient care. This study takes place in the SLaM Psychosis Clinical Academic Group.

### Sample

#### Inclusion criteria

Patients are eligible to participate if they are aged 18–65 years, have a primary clinical diagnosis of psychosis, are using specialist mental health services, are not currently in prison, speak and understand English, and, in the opinion of their key clinician, are sufficiently well to participate in a group therapy.

#### Exclusion criteria

Patients with serious cognitive impairment preventing meaningful participation in a group intervention, and those who in the opinion of their key clinician are unable to give consent or are too unwell to be interviewed.

### Sample size

A sample size of n = 30 per arm has been recommended for pilot studies to estimate location (mean) and variability (standard deviation) of candidate outcome measures to inform later sample size calculation [28]. WELLFOCUS PPT is conducted as a group intervention with about 8 participants in each group. We aim to conduct five waves of PPT groups versus control, each starting with 16 participants (n = 8 in the intervention and n = 8 in the control arm), giving a total sample of 80. Allowing for a drop-out rate of 20%, consistent with attrition in a feasibility study of a similar intervention with service users with psychosis [9], this gives an analysable sample of n = 32 per arm to generate estimates for well-being as our primary outcome. However, we will try to minimize dropouts in our study as suggested by Little et al. [31].

The Warwick-Edinburgh Mental Well-being Scale (WEMWBS) was shown to have a one-week re-test reliability of 0.83 [32]. The correlation between baseline and 3-months follow-up can be assumed to be below that. Assuming a correlation of 0.7 the power to detect an effect size of 0.5 on the WEMWBS at follow-up, adjusted for baseline, would be at 0.80. A more conservative estimate of a correlation between baseline and follow-up of 0.6 would allow us to detect the same effect size with a power of 0.71. Higher effect sizes would provide higher power. For example, an effect size of 0.6 could be detected with a power of 0.85 assuming a correlation between baseline and follow-up of 0.6. All power calculations are based on a significance level of 0.05 and were calculated using the STATA 12.

### Control group

Control group participants receive treatment as usual, consistent with the Care Programme Approach [33]. This includes systematic arrangements for assessing the health and social needs of people accepted into specialist mental health services, the formation of a care plan which identifies the health and social care required from a variety of providers, the appointment of a key worker to keep in close touch with the service user and to monitor and co-ordinate care, and regular review of and, where necessary, agreed changes to the care plan. Control group participants receive care from a multidisciplinary mental health team, and treatments may include medication, social or other individual or group-based psychological interventions. No psychological intervention based on positive psychology principles is currently provided.

### Intervention group

All intervention group participants receive treatment as usual, as described for the control group, and in addition receive WELLFOCUS PPT.

WELLFOCUS PPT is an 11-session intervention which aims to improve well-being in people with psychosis. All sessions begin and close with a music savouring exercise. Over the course of the 11 sessions, 10 exercises adapted from standard PPT are covered: positive introductions, savouring, good things, identifying personal strengths, personal strength activity, strength activity with significant other, forgiveness, one door closes another door opens, gratitude, and positive responding. All intervention exercises target at least one of the four target areas identified in the WELLFOCUS model. Every session develops an ‘ongoing exercise’, comprising an exercise to be completed, repeated, or reflected on in participants’ own time.

WELLFOCUS PPT is provided by two facilitators who follow the WELLFOCUS PPT manual (unpublished, on request from first author). Groups last approximately 90 minutes, with a 10 minute break in the middle. All facilitators have experience of facilitating therapeutic groups and of working with people with a diagnosis of psychosis. Facilitators are encouraged to show warmth, empathy, and genuineness in their interactions. Facilitators participate in all exercises themselves, they share personal examples from their own lives with the group, and are encouraged to do the ‘ongoing exercise’ in their own time. Participants are not prohibited from sharing distressing, unpleasant, or negative states and experiences in the group. Negative contributions are validated but not focused on. Instead, facilitators establish a link between the negative experience and one or more of the intervention’s target areas. Participants in the intervention group receive a phone call between each session to offer support with the ‘ongoing exercise’. All facilitators are invited to attend a monthly peer supervision meeting and we plan to report on the rate of attendance.

### Treatment fidelity strategies

We use principles for enhancing treatment fidelity outlined by the NIH Behavior Change Consortium [34], comprising strategies to increase treatment fidelity in five areas: study design, training providers, delivery of treatment, receipt of treatment, and enactment of treatment skills. Compliance with fidelity strategies will be assessed using checklists.

Fidelity strategies at the design level aim to ensure that a study can adequately test its hypothesis by ensuring the design is theory driven, and establishing procedures to standardise the dose and intensity, prevent contamination and deal with implementation setbacks. We used three fidelity strategies at study design level:

1. We use an evidence-based testable intervention model, i.e., the WELLFOCUS model.

2. To ensure the same treatment dose in all groups across the treatment arm, we send treatment reminders, work with fixed length group sessions, and a fixed number of mid-week telephone contacts per participant. We use a treatment manual which is followed in each group and provide the same information material to every participant.

3. To address possible implementation setbacks, we maintain a pool of trained facilitators, to avoid facilitator absence leading to group cancellations.

Fidelity strategies at the level of provider training aim to ensure adequate skill acquisition and skill maintenance of facilitators. We use three fidelity strategies at the level of provider training:

1. We provided two days of standardised training for all facilitators, led by the co-developer of standard PPT and the WELLFOCUS research team.

2. We provide monthly peer supervision sessions, which mirrors the group’s facilitator style and the focus on the four target areas as training boosters.

3. Following each session, both facilitators jointly write PPT-style notes on participants’ achievements in the four target areas, to avoid therapeutic drift.

Fidelity strategies at the level of treatment delivery aim to ensure provision of identical content across the individual groups in the intervention arm and to minimise contamination between arms. We use two fidelity strategies at treatment delivery level:

1. We manualised the intervention, using unpublished best practice guidelines (REMINDE – see http://www.equator-network.org/resource-centre/library-of-health-research-reporting/reporting-guidelines-under-development).

2. Facilitators met before each session to rehearse the session content as described in the manual, to minimise between-group differences.

Fidelity strategies at the level of treatment receipt aim to ensure that participants understand and perform the cognitive and behavioural skills delivered during treatment. This is an important and often neglected component of fidelity. If participants do not understand or are not able to implement the new skills during treatment, an otherwise effective intervention may be deemed ineffective. We use two fidelity strategies at the treatment receipt level:

1. To ensure participant comprehension we use concise and plain English information materials supported by pictures to avoid a focus on literacy.

2. The ‘ongoing exercise’ is supported and monitored by weekly calls and reviewed in the subsequent group session to increase both understanding and implementation of the involved skills.

Fidelity strategies at the level of treatment skills enactment aim to ensure that skills learned during the therapy are adequately applied in real-life settings. Enactment is different from treatment adherence and treatment efficacy. Treatment adherence relates to whether a participant performs a task definitive of a specific treatment (e.g., actually notes down a good thing that has happened) and treatment efficacy relates primarily to whether an intervention influences the hypothesised endpoint. Treatment enactment specifically relates to the extent to which a participant actually implements a new skill in their everyday life. It is possible that a study shows excellent enactment but poor adherence and poor efficacy. This would provide a good test of the intervention because the treatment skills are used but are not effective. By contrast, in a study with poor enactment neither adherence nor efficacy is likely to be high. We use two fidelity strategies at the level of skills enactment:

1. Participants are repeatedly reminded throughout the duration of the group of skills learned during past group sessions to encourage their ongoing application, e.g., savouring small things in everyday life or noticing good things and collecting them in a ‘good things’ box.

2. The last manualised session focuses on reflecting on the implementation of learned skills in everyday life.

### Measures

The primary outcome is the WEMWBS, a 14-item measure of positive well-being originally developed for the UK general population which frames well-being as a multi-dimensional construct [32]. The overall score is the sum of all items, varying from 14 (low well-being) to 70 (high well-being). The overall scale has proven feasible, reliable, and sensitive to change in people with various mental health problems, including psychosis [35]. Chronbach’s α for the scale lies between 0.87 and 0.91 and the one-week test-retest reliability at r = 0.83 [32,36].

Eight secondary outcomes are used.

#### Positive Psychotherapy Inventory (PPTI)

The PPTI consists of 25 questions rated on a 5-point Likert scale varying from “not at all like me” to “very much like me”. The overall score is the sum of all items, varying from 25 (low) to 125 (high). The scale contains five factors designed to assess the positive emotions, engagement, relationships, meaning, and accomplishment (each with a total score between 5 (low) and 25 (high)). It was specifically designed to capture changes due to PPT in clients [25]. The internal consistency of the scale was found at α = 0.80, its test-retest reliability at r = 0.81 in a sample of healthy participants [37].

#### Savouring Beliefs Inventory

The Savouring Beliefs Inventory is a 24-item scale that assesses individuals’ perceptions of their ability to derive pleasure through anticipating upcoming positive events, savouring positive moments in the present, and reminiscing about past positive experiences. It uses a 7-point Likert scale between “strongly disagree” and “strongly agree”. The overall score is computed by summing responses to the 12 positively-anchored items and subtracting responses to the 12 negatively-anchored items. Total scores can vary between −72 (low savouring beliefs) and +72 (high savouring beliefs) for the overall scale. Scores for the three subscales are calculated according to the same principle and can vary from −24 (low savouring beliefs on subscale) to +24 (high savouring beliefs on subscale). The overall scale showed an internal consistency of between α = 0.88 and 0.94 in different studies and a test-retest reliability of r = 0.84 [38]. It was also found feasible to use in people with psychosis [9].

#### Short Depression-Happiness Scale

The Short Depression-Happiness Scale measures affect on a bipolar continuum between depression and happiness [39]. It uses a 4-point Likert scale between “never” and “often”. The 6-item short version was found to have an internal consistency of between α = 0.77 and 0.92 in different studies, and a test-retest reliability of r = 0.68. The 3 positive items are reverse-scored which leads to overall scores varying from 6 (more depression) to 24 (more happiness). Preliminary normative data suggest that a score of 9 or less may be indicative of mild but clinically relevant depression [40].

#### Brief Psychiatric Rating Scale

The Brief Psychiatric Rating Scale is an 18-item observer-rated measure of psychiatric symptom severity [41]. It uses a 7-point Likert scale from “not present” to “extremely severe”. The overall score is the sum of all items, varying between 18 (low symptoms) and 126 (severe symptoms). The internal consistency of the overall scale lies between α = 0.65 and 0.79, its diagnostic sub-scales for withdrawal/retardation, thinking disorder, anxiety/depression, and activation vary from α = 0.766 to 0.879 [42,43]. Inter-rater reliabilities for this scale have been reported between 0.87 and 0.97. In terms of clinical interpretation, a total score of 31 was found to correspond to a clinical global impression rating of “mildly ill”, 41 to “moderately ill”, and 53 to “markedly ill” [44].

#### Integrative Hope Scale

The Integrative Hope Scale captures a comprehensive concept of hope and has been validated specifically for people with psychosis [45,46]. The scale contains 23 items that are rated on a 6-point Likert scale from “strongly agree” to “strongly disagree”. The 6 negatively anchored items are reverse-scored. The overall score is the sum of all items, varying between 23 (low hope) and 138 (high hope). The scale’s internal consistency lies at Cronbach’s α = 0.92 for the overall scale and the test-retest reliability at r = 0.84 in people with psychosis [45].

#### Rosenberg Self-Esteem Scale

The Rosenberg Self-Esteem Scale contains 10 items which are rated on a 4-point Likert scale from “strongly agree” to “strongly disagree”. The scale measures self-esteem by asking the respondents to reflect on their current feelings, with the 5 negatively anchored items being reverse-scored. The overall score is the sum of all items, varying between 0 (low self-esteem) and 30 (high self-esteem). Its psychometric properties have been repeatedly established in various client groups, including people with psychosis, and languages with the internal consistency found between α = 0.77 and 0.88, and the test-retest reliability between r = 0.82 and 0.88 [47,48].

#### Rogers Empowerment Scale

The Rogers Empowerment Scale is a 28-item instrument designed to measure subjective feelings of empowerment on a 4-point Likert scale varying from “strongly agree” to “strongly disagree”. After reverse-scoring of the 9 negatively framed items the sum of all items forms the overall score which can vary between 28 (low empowerment) and 112 (high empowerment). Its psychometric properties have been confirmed for people with psychiatric conditions, including psychosis, where the scale’s internal consistency was found at α = 0.86, and 6 of the 7 factors of the scale showed a re-test reliability of r >0.75 [49,50].

#### Sense of Coherence Scale

The Sense of Coherence Scale contains 29 questions to measure a person’s global orientation to view the world and the individual environment as comprehensible, manageable, and meaningful. It uses a 7-point scale Likert scale between “very often” and “very seldom or never”. Thirteen items are formulated negatively and have to be reversed for scoring. The overall score is the sum of all items, varying between 29 (low sense of coherence) and 203 (high sense of coherence). In different samples and translations, the scale showed an internal consistency of between α = 0.70 and 0.95, and a 1 year test-retest reliability from r = 0.69 to 0.78. [51].

In addition, five further measures are used. The Manchester Short Assessment of Quality of Life is an established 12-item measure that frames well-being in terms of subjective health-related quality of life and includes a specific item asking for satisfaction with life as a whole [52], which has been regarded as a measure of well-being in its own right [53]. The overall score is the mean of all item scores which can vary between 1 (low satisfaction with life) and 7 (high satisfaction with life). The scale’s internal consistence was found between α = 0.74 [52] and 0.81 [54] in different samples of people with mental illness. The Health of the Nation Outcome Scale is a widely used 12-item measure of social disability. Items cover a range of problem areas rated on a 5-point scale between 0 (no problem) and 4 (serious problem) with a resulting overall score of up to 48. Cronbach’s α for the scale varies between 0.59 and 0.76 [55]. The test-retest reliability was found to be mixed for all items, varying between r = 0.65 and 0.40 for seven items, and 0.31–0.32 for three items [56].

The Global Assessment of Functioning is an equally well accepted measure of functioning, which is used in a 2-item version assessing social and psychological functioning, each rated from 1 (most serious symptoms or disability) to 90 (no symptoms or disability) [57]. The items show an internal consistency of between α = 0.61 and 0.91, and a test-retest reliability of between r = 0.66 and 0.92 [58].

The CORE10 is a 10-item measure of symptoms. It is scored on a 5-point Likert scale from 1 “not at all” to 5 “most or all of the time” resulting in overall scores between 10 and 50. The scale’s internal consistency is α = 0.90 [59].

The Sociodemographics Form – Service User is a non-standardised measure modified from another RCT [60], which records the service user’s date of birth, gender, ethnicity, languages spoken, country of birth, education, employment, marital status, and housing. The rationale for inclusion of each outcome measure is shown in Table 1. Those in the intervention group additionally receive a process evaluation form at follow-up which asks to rate the effect of WELLFOCUS PPT on the hypothesised intervention model variables using a 10-point Likert scale.

**Table 1:** Outcome measures used in the WELLFOCUS study

**WELLFOCUS model component**	**Measure**	**Rater**	**Rationale**
**Distal outcome**			
Personal well-being	WEMWBS	Patient	Measures overall well-being, i.e., enhanced sense of self
**Proximal outcomes**			
Good feelings	Savouring Beliefs Inventory	Patient	Assesses pleasure in the past present and future, which is a form of good feeling addressed in the intervention
	PPTI positive emotions	Patient	Assesses enjoyment and happiness, which is a form of good feeling addressed in the intervention
Symptom relief	Short Depression-Happiness Scale	Patient	Measures the reduction of depression, which is an intended outcome of PPT
	Brief Psychiatric Rating Scale	Researcher	Measures general symptom severity including psychosis-specific symptoms
	CORE10	Patient	Measures general symptom severity
Connectedness	PPTI relationships	Patient	Measures the presence of supportive relationships as a form of connectedness addressed in the intervention
Hope	Integrative Hope Scale	Patient	Measures hope, which is an indicator of well-being
Self-worth	Rosenberg Self-Esteem Scale	Patient	Measures self-worth, which is an indicator of well-being
Empowerment	Rogers Empowerment Scale	Patient	Measures empowerment, which is an indicator of well-being
Meaning	Sense of Coherence Scale	Patient	Measures meaning, which is an indicator of well-being
	PPTI meaning		Provides a PPT-specific measure of meaning
**Other outcomes**			
Quality of Life	Manchester Short Assessment of Quality of Life	Patient	Quality of life is a form of well-being measure, allowing triangulation with the WEMWBS
Social disability	Health of the Nation Outcome Scale	Researcher	To give opportunity to compare changes in well-being with social disability
Functioning	Global Assessment Functioning	Researcher	To give opportunity to compare changes in well-being with functioning

PPT, Positive Psychotherapy; PPTI, Positive Psychotherapy Inventory; WEMWBS, Warwick-Edinburgh Mental Well-Being Scale.

### Fidelity assessment

Corresponding with the strategies to improve treatment fidelity, we use fidelity measures at three of the five levels of fidelity, i.e., study design, provider training, and treatment delivery. The other two levels of fidelity – treatment receipt and skills enactment – will be assessed as part of the process evaluation.

To assess fidelity at the study design level, deviations from session lengths and the length of mid-week calls is recorded (to establish treatment dose) and group cancellations and any changes to group times are recorded (to identify implementation setbacks). To assess fidelity at the provider training level, facilitators’ presence at training and supervision sessions is recorded. To assess fidelity at the treatment delivery level, adherence to treatment protocol is rated by the co-facilitator for each session on a fidelity evaluation scale developed for WELLFOCUS PPT.

### Process evaluation

The aim of the process evaluation is to test the intermediate processes outlined in the WELLFOCUS model between the intervention (tested by fidelity assessment) and outcome. Process evaluation strategies include ratings and notes taken by the co-facilitators directly after each session and phone call for each participant and each week, and additional qualitative interviews and focus groups after the end of the therapy.

The co-facilitator collected process evaluation includes five assessment strategies at treatment receipt and skills enactment level which map onto the WELLFOCUS intervention model:

1. Presence of participants in group sessions

2. Engagement of participants in exercises during treatment groups and a facilitator rating of whether this was perceived as beneficial

3. Facilitator rating of behavioural intent to undertake the ‘ongoing exercise’, based on the session in which the ‘ongoing exercise’ was set

4. Facilitator rating of engagement and benefit from the ‘ongoing exercise’, based on the session in which the ‘ongoing exercise’ was reviewed

5. Length and content of the weekly telephone calls.

Qualitative process evaluation comprises individual interviews and focus groups with trial participants, and individual interviews with group facilitators. Participants will be asked to take part in either an individual interview (50%) or a focus group (50%).

Semi-structured interviews will ask about participants’ experience of taking part in the study, how the intervention was delivered, and what impact the intervention has had on their well-being and life generally. Questions will also relate to the WELLFOCUS model and processes facilitated by the therapy. Suggestions for further improving the intervention and trial processes will be collected. As far as possible, those who dropped out of the intervention arm or attended irregularly will be asked for reasons including contextual factors influencing attendance and for suggestions to improve attendance rates in the definitive RCT. Focus groups will also allow discussion of any suggestions for improvements of the intervention. Hence, topics that come up in interviews, particularly about problematic issues in relation to therapy content, delivery, and study procedures, will feed into focus groups.

All clinicians involved in providing the therapy will be interviewed to obtain feedback on the WELLFOCUS manual, the intervention delivery, and the trial procedures.

### Trial procedures

All researchers are trained in the use of all standardised outcome assessments and in semi-structured interviews or focus groups for the process evaluation. Ongoing supervision is provided from experienced clinical researchers.

### Recruitment and randomisation

The study is introduced to all staff members in the participating teams. Care coordinators, key nurses or other appropriate staff members are asked to identify potentially eligible service users from their case load. Potentially eligible participants receive information about the study from the clinician. In addition, information about the study is sent to eligible service users on two research registers (which involves consent to be contacted by researchers). These are followed-up for recruitment via telephone by the research team. An appointment is scheduled for assenting participants from all sources with a member of the research team to provide further information on the study. In this meeting, the study is explained, written information is provided, and an opportunity is given to ask any questions. If the participant wishes to have time to consider participation, a second meeting is scheduled. Once the participant has given informed consent, baseline assessments are completed. Participants receive an ID number which is entered in the web-based randomisation system together with age (for validation). Some teams may have fewer than 16 eligible participants, and the minimum number of participants will be 8 (i.e., 4 per arm), with block for randomisation. Where possible we will over-recruit to a maximum of 20 participants in other teams. When participants from a participating clinical team have all been entered into the database, randomisation is conducted. Randomisation involves independent allocation to one of the two trial arms on a 1:1 basis. The use of stratification was considered disproportionate for a pilot RCT. The generation and implementation of the randomisation sequence is conducted independently by the King’s Clinical Trials Unit (registration number 053).

### Assessment

Outcomes are measured pre-randomisation and at the end of the intervention (i.e., 3 months after baseline). Every effort will be made to include patients who drop out of treatment in the follow-up assessments to enable intention-to-treat analysis [30]. Process and fidelity measures are collected by the co-facilitators for each participant after each group session. Qualitative process evaluation is conducted with those in the intervention arm, who are asked to participate either in an individual interview or in a focus group (with alternating allocation based on finishing order of groups). Participants are offered £20 as compensation for their time after completing each assessment and either the interview or focus group (i.e., £60 in total for intervention, £40 for control). The £20 difference in payment between the groups might increase follow-up rates for the process evaluation data from intervention group participants, but remuneration for the outcome evaluation is the same for both groups.

### Approaches to minimise bias

It is not possible for service user participants or facilitators to be blind to allocation status. The resources needed to ensure blinding in research assessors would be disproportionate in a pilot study; therefore, this trial is unblinded. However, several approaches will be used to minimise bias.

#### Addressing bias at participant selection

This study uses a convenience sample of service users considered by team leaders to be fit to participate. This may lead to selection bias. However, internal validity is protected due to the random allocation strategy and not revealing the block size.

#### Addressing bias at allocation

All randomisation is undertaken by the independent King’s Clinical Trials Unit.

#### Addressing bias in baseline data

Baseline data are collected before allocation, to reduce assessment bias due to inadequate concealment.

#### Addressing bias in the intervention

Fidelity to the WELLFOCUS manual is monitored to ensure comparability of the intervention between local groups.

#### Addressing bias in follow-up data

All participating services users are followed-up and included in the analysis using an intention-to-treat approach to reduce the impact of selective attrition.

#### Addressing bias in outcome data collection

Bias in the outcome data is minimised by the use of standardised objective assessments and by rater training and supervision. Post-treatment outcomes are, where possible, assessed by researchers who were not involved in the participant’s baseline assessment or their group.

#### Addressing bias in process evaluation

As far as possible the focus group and interview data are collected by a researcher not involved in delivering the intervention to the respective participants, to minimise social desirability bias.

#### Addressing bias in analysis

No data regarding the allocation status are stored in the data entry database, following Good Clinical Practice [61]. The primary data analysis is undertaken blind to allocation status*.* Allocation status is coded as A and B in all data requests.

### Data handling

All study data will be input by the research workers who collected the data. Rigorous approaches to validating and verifying the data will be used, including rater training to achieve an acceptable concordance in administering standardised assessments, development of a database with allowable values for variables as a validation check, double rating for 10% of the data to identify transcription errors, and data cleaning and preliminary analysis led by a statistician to identify invalid responses, checking for outliers, and unexpected patterns in the relationships between variables.

Protected data storage with clear access protocols in line with Good Clinical Practice Guidelines is used to store allocation and outcome data separately. All data are stored in either a locked filing cabinet or in an electronic password-protected database.

### Analysis plan

All qualitative data will be audio taped, fully transcribed, and quality checked. Transcripts and notes will be subjected to rigorous thematic analysis [62]. Results from the qualitative analysis of process evaluation data will be integrated with results from quantitative statistical analysis to address the three study objectives. Overall, the results of this pilot study will inform the design of a future definitive RCT.

#### Objective 1: Piloting the intervention

Acceptability of the intervention will be assessed by statistically exploring compliance patterns. This will involve the calculation of patterns of adherence, i.e., attendance rates, completion rates, and proportion of homework completed. Descriptive statistics will be used to evaluate whether levels of adherence and variability in adherence depend on therapy group and basic participant characteristics, such as gender, age, and symptom severity. Rate of loss to follow-up and its dependence on adherence level and on basic participant characteristics will be explored. The quantitative results will be combined with results from the qualitative process evaluation to suggest recommendations for specific adaptations to the intervention components and delivery. Qualitative and quantitative measures will be used to investigate whether the processes and changes subjectively experienced by participants correspond with the processes outlined in the WELLFOCUS model.

#### Objective 2: Piloting the trial processes

Descriptive statistics will be used to assess recruitment rates in relation to referrals received by clinical staff. Mean time between referral and consent will be calculated. Attrition between referral, consent, and randomisation will be described. Waiting times will be analysed in relation to attrition rates. The proportion of those receiving the intervention as allocated will be calculated. Those receiving and not receiving the intervention will be compared on participant characteristics. Qualitative data will supplement quantitative data to explain discrepancies between allocation and receipt of intervention. Reasons for facilitator drop-out will be explored in the qualitative analysis. Qualitative data from clinician feedback and quantitative data on recruitment rates will be used to formulate recommendations for adapting recruitment procedures, eligibility criteria, participant information, and seeking consent. Any issues arising with regards to randomisation procedures from the qualitative process evaluation and researcher feedback will be used to suggest improvements. Feedback from researchers and participants will be used to suggest improvements to the feasibility of data collection forms.

#### Objective 3: Piloting the evaluation strategy

The usefulness of fidelity and process evaluation approaches will be determined by exploring whether researcher ratings of fidelity to the manual and process factors, such as engagement and beneficial effect of exercises, helps to improve prediction of outcome and future attendance as compared to recording group attendance only. The confidence interval approach will be used to help determine the suitability of the outcome measures and to inform the sample size calculation for a definitive RCT. This approach involves estimating the confidence interval of the difference between intervention and control group at follow-up, adjusted for baseline. The inclusion of zero (i.e., point of no effect) and the inclusion of a clinically relevant effect into the confidence interval will help to estimate the suitability of measures for a definitive RCT [28]. Further considerations to be taken into account for the choice of outcome measure for the definitive RCT will include the acceptability of the scale (as measured by the number of missing responses), the magnitude of correlation between baseline and follow-up on this scale, and the overall variance of the scale. The group means, standard deviations, and correlations between baseline and follow-up will be used to inform the sample size calculation for the definitive RCT.

In addition, outcomes will be analysed to estimate effectiveness of the intervention. This analysis will be done on an intention-to-treat basis, with participants analysed in the group to which they were randomised irrespective of their compliance with the assigned trial arm. To estimate effectiveness of the intervention, WEMWBS (primary outcome) at pre-treatment (baseline) and 3-month follow-up will be analysed using a random effects regression of outcome on trial arm adjusted for baseline score including a random effect to allow for between group variations in the intervention arm. Where relevant, we will also consider using other approaches to address missing data such as complier-average causal effect approaches [63].

Divergence of normality will be assessed for the distribution of all variables as will linearity of correlations and equality of variance. The choice of statistical methods will be adapted accordingly. *P* values of 0.05 will be considered significant in all cases. All analyses will be carried out in SPSS Version 20 and Stata Version 12.

### Trial management

Prof Mike Slade (principal investigator; PI) has overall responsibility for the trial. The Trial Manager is Dr Beate Schrank, who is responsible for co-ordination.

### Risk and adverse events

Relevant trust policies relating to potential areas of risk, such as risk management and medication, will be adhered to. Serious adverse events will be monitored by the Trial Manager, who will report these to the PI and where there is a possibility that they are linked to the trial, the Trial Steering Committee will be informed.

### Trial supervision

An independent Trial Steering Committee (TSC) has been convened, with membership comprising Prof Stefan Priebe (Professor of Social Psychiatry, Queen Mary and Westfield College, University of London) (Chair), Jan Wallcraft (independent user researcher), and Michael Wright (funder representative). At the first TSC meeting the need for a Data Monitoring and Ethics Committee, interim analyses, and stop rules for the trial were discussed. Any serious untoward incident is reported to the TSC Chair.

### Data handling and record keeping

The research workers will enter data into a secure password-controlled database. Data entry will include validation checks.

Interim analysis will be performed, as requested by the TSC and as needed by the PI to monitor progress. The only documentation which will contain identifying material are the participants’ contact details and consent forms, which will be stored separately from other study data with linkage through an ID number. Any audiotape recordings will be destroyed once the transcription has been checked for accuracy. All paper forms of these data will be stored in locked filing cabinets at the Institute of Psychiatry. Only the research team will have access to these filing cabinets. All other data will be identified by a participant identification number only. A file linking the participant identification number and personal data will be password-protected and stored on a secure server at the Institute of Psychiatry. Only the research team will have access to these data. Electronic and paper data will be retained for 10 years. All members of the study team will receive MRC Good Clinical Practice training in RCTs and we shall follow Research Governance arrangements.

### Data access

This study will generate qualitative data comprising interview transcripts and associated analyses, and quantitative data from questionnaires and process evaluation forms. Exclusive use for primary research by the research team is envisaged for no more than 3 years following the study, to meet dissemination goals. Both the quantitative and qualitative data will be shared in anonymised form only. It is anticipated that the data may be used for secondary re-analysis as well as contributing to larger datasets of routinely collected outcome data. Archiving and curating (including data sharing agreements and management of access rights) will be undertaken within the framework used by King’s College London, with due attention to issues of ethical (including consent and confidentiality aspects), legal, and institutional regulatory permissions.

### Reporting of trial

The trial data will be reported in line with the extension of CONSORT guidance for trials assessing non-pharmacological treatments and pragmatic trials [64-66].

### Publication

The results of the research will be targeted for publication in peer-reviewed journals of general and special interest, and presented at international conferences. Lay summaries of the results will be published on the research section webpage (researchintorecovery.com).

## Trial status

Recruitment for the trial began on 27 March 2013 and is on-going.

## Abbreviations

MRC: Medical research council; PI: Principal investigator; PPT: Positive psychotherapy; RCTs: Randomised controlled trials; SLaM: South London and Maudsley NHS foundation trust; TSC: Trial Steering Committee; WEMWBS: Warwick-Edinburgh Mental Well-being Scale.

## Competing interests

The authors declare that they have no competing interests.

## Authors’ contributions

BS: Conception and design, data collection and analysis, manuscript writing and final approval of the manuscript. SR: Data collection, critical revision, and final approval of the manuscript. TC: Critical revision and final approval of the manuscript. TR: Critical revision and final approval of the manuscript. AT: Conception and design, critical revision, and final approval of the manuscript. MS: Conception and design, supervision of data collection and analysis, supervision of manuscript writing, and final approval of the manuscript.
